# Recent Warming of Lake Kivu

**DOI:** 10.1371/journal.pone.0109084

**Published:** 2014-10-08

**Authors:** Sergei Katsev, Arthur A. Aaberg, Sean A. Crowe, Robert E. Hecky

**Affiliations:** 1 Large Lakes Observatory, University of Minnesota Duluth, Duluth, Minnesota, United States of America; 2 Department of Physics, University of Minnesota Duluth, Duluth, Minnesota, United States of America; 3 Department of Earth, Ocean, and Atmospheric Sciences, University of British Columbia, Vancouver, British Columbia, Canada; 4 Department of Biology, University of Minnesota Duluth, Duluth, Minnesota, United States of America; University of Vigo, Spain

## Abstract

Lake Kivu in East Africa has gained notoriety for its prodigious amounts of dissolved methane and dangers of limnic eruption. Being meromictic, it is also expected to accumulate heat due to rising regional air temperatures. To investigate the warming trend and distinguish between atmospheric and geothermal heating sources, we compiled historical temperature data, performed measurements with logging instruments, and simulated heat propagation. We also performed isotopic analyses of water from the lake's main basin and isolated Kabuno Bay. The results reveal that the lake surface is warming at the rate of 0.12°C per decade, which matches the warming rates in other East African lakes. Temperatures increase throughout the entire water column. Though warming is strongest near the surface, warming rates in the deep waters cannot be accounted for solely by propagation of atmospheric heat at presently assumed rates of vertical mixing. Unless the transport rates are significantly higher than presently believed, this indicates significant contributions from subterranean heat sources. Temperature time series in the deep monimolimnion suggest evidence of convection. The progressive deepening of the depth of temperature minimum in the water column is expected to accelerate the warming in deeper waters. The warming trend, however, is unlikely to strongly affect the physical stability of the lake, which depends primarily on salinity gradient.

## Introduction

Deep meromictic lakes are good climate monitors, as changes in heat fluxes across the lake surface become reflected in heat content of the deeper waters [1]. Consistent with recent atmospheric warming, surface temperatures of stably stratified East African Great Lakes increased by about one degree over the last century [1], [2]. The temperature rise could be linked to a number of adverse effects, such as increased physical stability of the water column that decreases nutrient upwelling and diminishes ecosystem productivity [1], [2].

**Figure 1:**
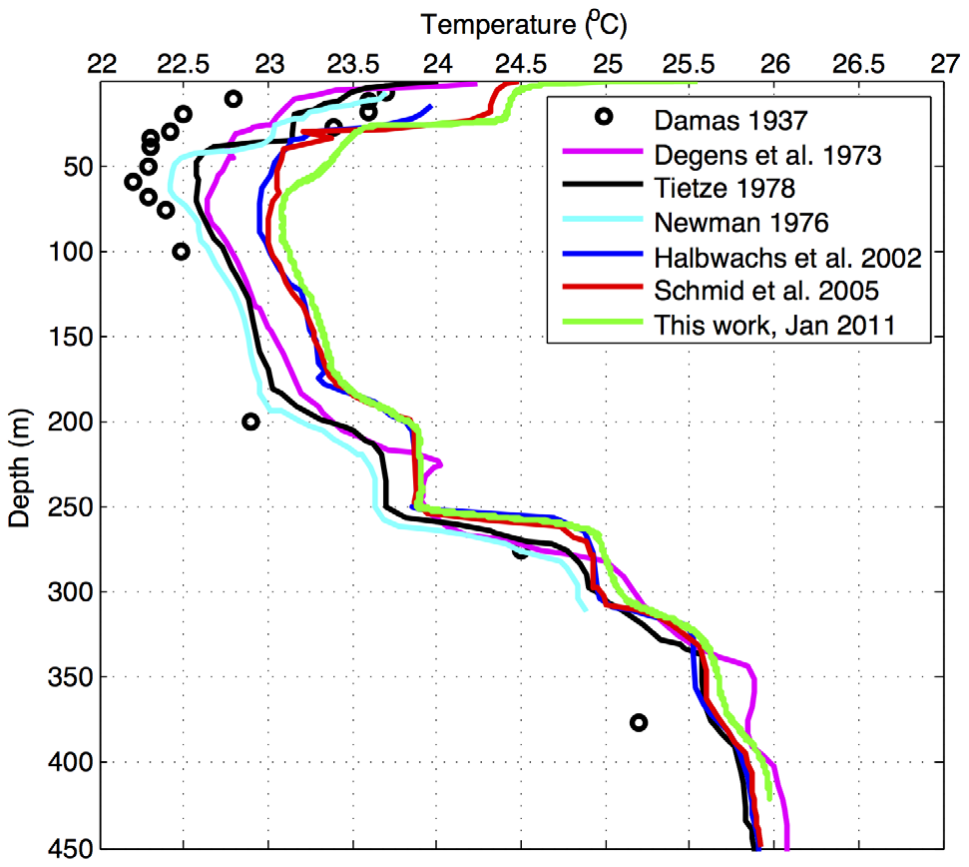
Evolution of temperature profiles in Lake Kivu.

The 450 m deep Lake Kivu, at the border of Rwanda and the Democratic Republic of the Congo, is unique among the African Great Lakes in that in addition to heat exchanges with the atmosphere its temperature is affected by subterranean heat inputs. The sublacustrine heat comes from a complex geological system that includes two active volcanoes, Nyiragongo and Nyamuragira [3]–[5], which constitute the lake's northern watersheds. These deep heat sources cause the lake temperature to increase with depth below the surface mixed layer, a feature not observed in other lakes. In addition to these unique features, the lake is best known for the prodigious amounts of carbon dioxide and methane in its deep waters [6], [7], which present both a hazard of a catastrophic limnic eruption [8], [9] and an economic opportunity of extracting the methane for energy generation. Persistent stratification in Lake Kivu is maintained by a salinity gradient with multiple pycnoclines, the strongest ones located at the depths of 60, 160, 250, and 310 meters. The pycnoclines are believed to be maintained by several sublacustrine inflows, which preserve the salinity and temperature gradients against dissipation by diffusion [8]. The uppermost pycnocline (60 m) marks the lower boundary of the epilimnion and the seasonal maximum depth of wind-induced mixing. The temperature decreases from surface into the epilimnion and down to the depth of approximately 80 m, but increases below that depth due to sublacustrine heat inputs (Fig. 1). The resultant reverse temperature gradient in deep waters implies an upward transport of heat towards the depth of the temperature minimum. This heat may be removed only through epilimnetic mixing, e.g. if weaker temperature gradients during a dry season [7] allow mixing to that depth, or if a cold inflow enters the lake at that depth.

Past studies [10] have reported surface warming of Lake Kivu of up to 0.5°C over the past 30 years. Monimolimnion warming, on the other hand, has received less attention. Until recently, warming was thought to be dominated by the atmospheric signal and limited to upper 250 m (the depth of the strongest pycnocline [4], [10]). Thermal effects of the Nyiragongo lava inflows after a 2002 eruption were deemed insignificant [4], [10]. Recently, however, warming below 250 m was also acknowledged [11], though whether the source of the heat was atmospheric or subterranean was not identified. Constraining the rate of the heat transfer in the deep monimolimnion is difficult [12], as the reverse temperature gradient results in double-diffusion structures [13] that are intermittent and often laterally localized within several hundred meters [12]. Non-diffusive transport mechanisms, such as convection generated by localized heat sources or density flows [14] have not been quantified. Despite several attempts to calculate mixing coefficients and fluxes [12], [15], [16], the rates of vertical mixing remain uncertain.

In this paper, we compile multi-decadal records of lake temperature profiles that show that the temperatures have increased throughout the entire water column of Lake Kivu. We quantify these increases, as well as the extent of seasonal mixing and epilimnetic heat removal. Focusing on multi-year and multi-decadal trends, we discuss the likely causes of warming, sources of heat in the surface mixed layer and deep waters, evidence for convective mixing in deep waters, and implications for lake stability.

## Methods

Depth profiles of temperature and conductivity were taken in Lake Kivu using a Sea&Sun CTD 90 M probe. The manufacturer's stated accuracy was 0.002°C and precision was 0.0002°C. A string of Onset U22 temperature recorders was deployed on a rope suspended from the KP1 methane extraction platform (1°43′ 56.445″ S, 29°14′ 34.246″ E) from January 2011 to March 2013. The temperature recorders had a precision of 0.02°C, which was sufficient for the purpose of resolving temporal temperature variations at their respective depths. Systematic errors within the recorders' accuracy range (0.2°C) were corrected by comparing their readings with those taken by the CTD. The deployment was verified in November 2011 and October 2012, at which point several faulty recorders were replaced and additional ones installed. A titanium-cased Seabird (SBE39) thermistor (accuracy 0.002, precision 0.0001°C, stability 0.0002°C/month) was deployed at the depth of 355 m on a rope suspended from the Rwanda Energy Corporation (REC) platform (1°43′ 55.9″ S, 29°14′ 34.1″ E) between September 2012 and March 2013. A second SBE39 temperature recorder equipped with a pressure sensor was deployed at 68 m depth to monitor for possible changes in deployment depth due to displacement of the platform or elasticity of the suspended rope. Historical temperature profiles in Lake Kivu water column over the past 80 years were obtained by digitization from published literature sources. The pressure-to-depth conversion for the CTD profiles was performed by multiplying the gauge pressure in dbar by 1.0197, consistent with the conversion used in previous studies in Lake Kivu [17]. This ignores the difficult-to-calculate depth variation of water density in Lake Kivu [4] but results in an error of no more than 0.5%, which is on the order of the CTD's pressure sensor accuracy. Water samples were taken from a selection of depths in both the main basin (in 2011 and 2012) and Kabuno Bay (in 2012) using a Niskin bottle with the pressure valve loosened to allow the exsolving gasses to escape on ascent. The samples were placed in crimped vials with no headspace by overfilling them from the bottom using a tube connected to the Niskin bottle spigot, and subsequently analyzed for the ^18^O and ^2^H isotopic composition at the GEOTOP facility at McGill University. Additional samples were collected by hand from a surface stream (1° 42' 14.99" S, 29° 16' 14.71" E), a hot spring near Gisenyi, and a single rain event near Kabuno Bay (1° 38' 28.63" S, 29° 8' 18.56" E, October 1, 2012). Duplicate water column samples taken according to USEPA protocols were analyzed for CFC concentrations by the Tritium Laboratory at the University of Miami, who also calculated the recharge ages (effective time of last contact with the atmosphere, in years before sampling) according to standard procedures. Analytical data have been made publicly available through the IEDA EarthChem data repository. Data are also available upon request to the corresponding author. Research in Rwanda was conducted under permit MINEDUC/S&T/0011/2010.

## Results

### Stratification and mixing

**Figure 2:**
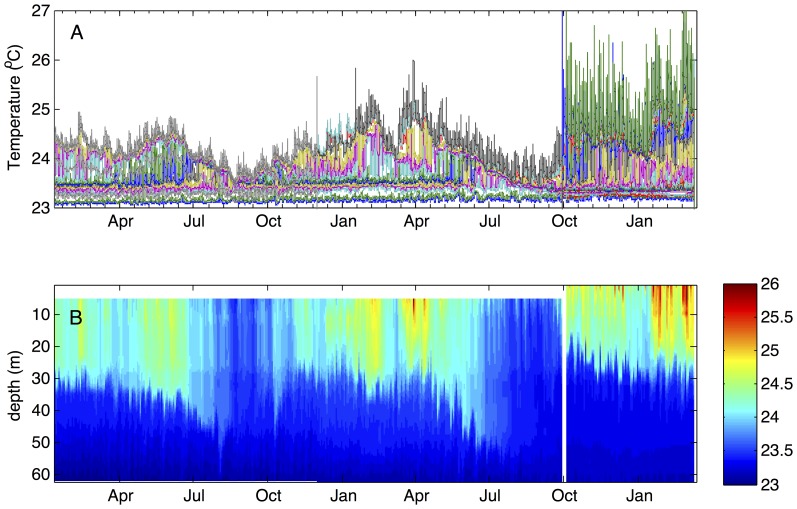
Epilimnion temperatures between January 2011 and March 2013. Temperatures were recoded by a string of moored Onset temperature loggers. A. Records from individual thermistors. B. Same data as a contour plot against depth. Thermistors were recovered and redeployed in November 2011 and October 2012, at which times several loggers were replaced and additional ones added.

**Figure 3:**
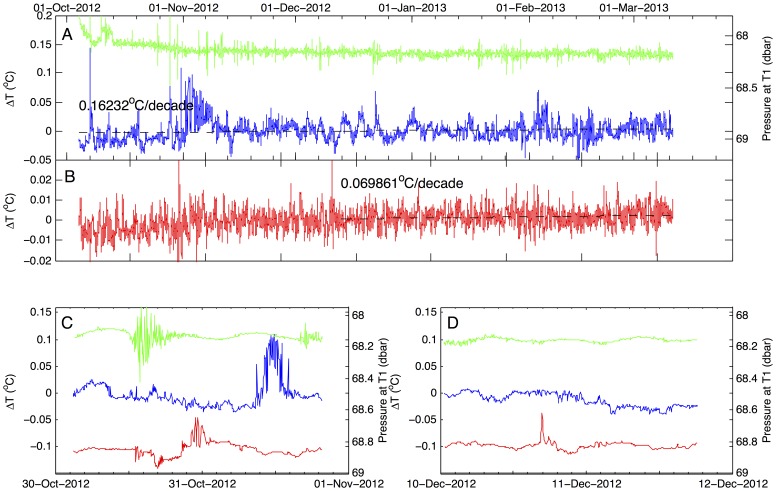
Deep-water temperatures between October 2012 and March 2013. Temperatures were recorded using moored Seabird temperature loggers. A,B: Temperature time series (deviations from the mean) at the depths of 68 m (T1) and 355 m (T2). The green line is pressure recorded at T1. C,D: The same but in more detail for two anomalous events in October and December 2012.

The temperature (Fig. 1) and conductivity (not shown) distributions in Lake Kivu conform to the salinity-controlled stratification described in multiple previous studies (e.g. [8]). The temperature minimum was observed in 2011–2012 at about 78 m depth (Fig. 1). Excursions in temperature and salinity profiles (not shown here) and comparison of profiles taken at different locations indicated cold sublacustrine inflows at depths 166, 173, and 242 m, and a warm inflow between 260 and 300 m depth, consistent with previous findings [10], [18]. Double diffusion “staircase” structures in temperature profiles were detected at depths intervals 170–200 m and 275–340 m. Temperature records in the epilimnion (Fig. 2) indicated that surface mixing during the dry season in 2011 and 2012 reached the depth of approximately 55 m, about 20 m shallower than the depth of the temperature minimum. The temperatures below the mixing depth, recorded by thermistors, increased at an average rate of ∼0.02–0.04°C per year (*p*>0.05) (Fig. 2). The temperature increase recorded by a more precise Seabird recorder at 68 m depth between October 2012 and March 2013 was 0.016°C per year (*p*>0.05) (Fig. 3).

The temperature time series recorded by the deep-water thermistor at 355 m depth shows temperature variations on the order of 0.01°C. This variance is characteristic of the temporal variability in CTD profiles at this depth, though greater than the variability reported in the deep waters of Lake Matano, a 700 m deep meromictic lake in Indonesia with no known heat sources at depth [19]. Importantly, the time series in Fig. 3 reveals several events with substantially larger temperature excursions, on the order of 0.05°C.

### Water isotopic signals

**Figure 4:**
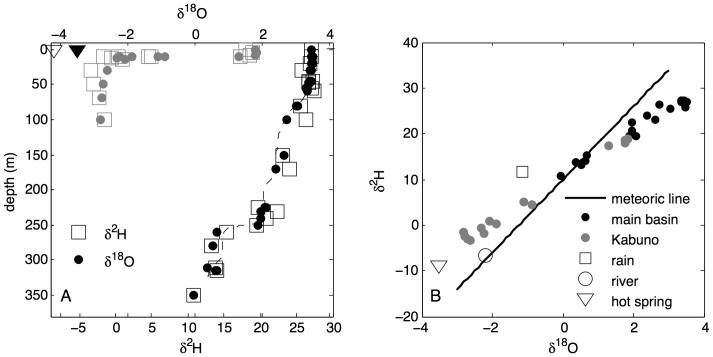
^18^O and ^2^H isotopic signals in Lake Kivu's main basin (black) and Kabuno Bay (grey). Also shown are data for a surface hot spring in Gisenyi (triangles), surface stream in Gisenyi (open circle), and a single rain event (square in panel B). The dashed line in panel A is an inverted and scaled profile of water conductivity, shown here for illustration as an indicator of water column stratification.

The ^18^O and ^2^H isotopic composition (Fig. 4) of Lake Kivu water column falls on a line between two endmembers: surface water where isotopic composition is dominated by the evaporative signal, and deep water where the isotopic signature trends towards the composition of groundwater, as exemplified by the composition at a hot spring in Gisenyi. The waters of Lake Kivu become isotopically lighter with depth, generally following the depth dependence of water density/conductivity (Fig. 4), which suggests that the depth distributions of isotopes are controlled by mixing rates (e.g. [19]). The waters in Kabuno Bay, which is separated from the main basin by a shallow sill, differ radically in their isotopic composition from the main basin. Water below the permanent pycnocline at 10 m in Kabuno Bay is isotopically much lighter than anywhere in the main basin (Fig. 4) but falls along the same line between the evaporative and groundwater endmembers (Fig. 4). This suggests that Kabuno Bay, being smaller, is more affected by sublacustrine inflows, many of which are believed to be located along the north shore of Lake Kivu. Small-scale variations in the depth distribution of ^18^O isotopes vs. ^2^H isotopes may reflect the inflow compositions. For instance, the δ^2^H profile in Kabuno Bay exhibits a minimum near the persistent pycnocline, at the depth where a cold inflow along the north shore has been detected in our CTD profiles (not shown). Similarly, Fig. 4 suggests inflows in the main basin at around 230 m, 260 m, 170 m, 70 m, and 35 m depth. The trends observed in our data are significantly clearer than those presented previously [5], due to higher spatial and concentration resolutions.

CFC concentrations in the monimolimnion were low. At the depth of 80 m, which approximately corresponds to the temperature minimum, the recharge age was>40 years, indicating that surface mixing did not reach that depth in recent past.

## Discussion

### The warming trend

**Figure 5:**
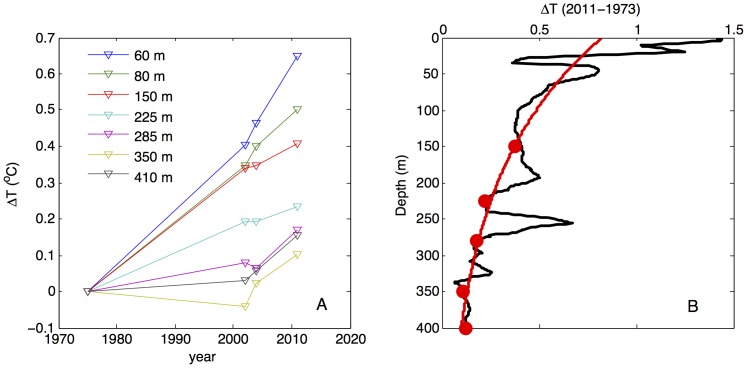
Changes in water column temperature. A) At selected depths against time, relative to 1975 measurements [32]. B) As a function of depth, based on comparison of our January 2011 profile with [32]. The exponential fit line is drawn through points (marked by symbols) between major thermoclines where temperature gradients are minimal and also least affected by the vertical movement of thermoclines.

A compilation of previously published and recently obtained temperature profiles (Fig. 1) reveals that temperatures in Lake Kivu have been increasing throughout the entire water column. Temperatures at the depth of temperature inversion (∼78 m) have increased by approximately 0.5°C since mid-1970s, a change of ∼0.12°C per decade. This is matched or exceeded by the warming rates (∼0.2°C per decade) recorded by our instruments below the depth of seasonal mixing in 2011–2012 (Figs. 2–3). Temperatures in the upper epilimnion, averaged over seasonal variations, seem to have increased over the past 40 years by half a degree to a degree (Fig. 1). Waters below 350 m have warmed since the 1970s by about 0.15°C (Fig. 1), though this number is less certain as early profiles exhibit significant variability (Fig. 1). It is worth noting that the profiles by Degens (1973) [20] and Newman (1976) [16] were taken at about the same time, on the same cruise. Degens's modified heat flow recorder likely did not have the same response time and accuracy as Newman's calibrated and rapid thermistors, so Newman's profile is likely to be more accurate. That this profile is similar and only slightly offset from the data of Damas (1937) [21] suggests that most of the warming (at all depths) occurred in the last 40 years. Figure 5b shows the evolution of temperatures at selected depths between major thermoclines where temperature gradients are minimal. The monimolimnion warming is strongest near the mixolimnion and progressively decreases with depth (Fig. 5). The warming rates inferred for the deep monimolimnion (Fig. 5) are consistent with the trend seen in our thermistor data (Fig. 3). Concurrent with warming, the thermal structure of the lake has changed in that the major thermoclines (pycnoclines) moved upward by about 15 m since the 1970s (Fig. 1; *see* also [14] and [18]), likely as a result of subsurface water inflows. Interestingly, the thermocline upwelling velocities (about 0.1 m y^−1^ for the 370 m thermocline, 0.2 m y^−1^ for the 310 and 250 m thermoclines, and 0.35 m y^−1^ for the 160 m thermocline) roughly match the values for the vertical advection velocities in the “steady state” model of [8] (from 0.15 m y^−1^ below 250 m to around 0.79±0.13 m y^−1^ between 110 and 200 m depth; cited in [22]).

The warming trend in the mixed layer of Lake Kivu is consistent with changes in other large African Lakes. In Lake Malawi, temperatures at 100 m depth are increasing at a rate of 0.06±0.02°C per decade [23]. In Lake Tanganyika, the upper water-column (150 m) has been warming at 0.1±0.01°C per decade since 1913, and deep waters (600 m) by ∼0.05 C per decade since 1938 [2], [24]. Lake Victoria warmed by 0.3°C between 1960s and 1991 (0.1 C per decade [25]), and Lake Albert warmed by 0.5°C between 1963 and 1990s (∼0.15°C per decade [13]). Warming typically results either from an increased amount of absorbed heat (e.g. due to higher air temperatures or reduced cloud cover) during the wet season or a decrease in evaporative cooling (e.g. due to an increased air humidity) during the dry season. Long-term trends in air temperatures around Lake Kivu are poorly known. Across the region, however, air temperatures kept pace with the global increase of 0.6±0.2°C over the past century (∼0.07 per decade [26]): temperatures increased by ∼0.5°C since 1980 in Kenya (0.15°C/decade [27]), 0.4°C since 1930s in winter around Lake Malawi (0.05°C/decade [23]), and 0.5–0.7°C between 1950 and 1995 in Lake Tanganyika (∼0.13°C per decade [2]). The Berkeley Earth compilation of meteorological data suggests the warming rate of 0.22°C/decade for Rwanda since the 1970s.

### Heat transport, mixing, and causes of warming

The reverse temperature gradient below 80 m depth implies an upward transport of heat from deep waters towards the depth of temperature inversion. The epilimnetic heat fluxes are also directed (downward) towards this depth. Though epilimnion temperature gradients become weaker during the dry season [7], mixing does not normally reach the T inversion depth (Fig. 2), although occasional deeper mixing events have been suggested [18]. The total increase in the main basin's heat content observed below 80 m between 1973 and 2011 (∼200 MJ m^−2^) requires an average excess heat flux through the uppermost chemocline of ∼0.18 W m^−2^. In contrast to the main basin of the lake, the temperature profiles in Kabuno Bay exhibit a strong temperature minimum throughout the year [7], and the temperature minimum is maintained by a cold inflow at that depth (10 m) (Fig. 4 and unpublished temperature data). In the main basin, cold inflows at the depth of the temperature minimum have not been detected and a calculation based on the balance of heat fluxes also suggested the absence of such an inflow [17].

Temperature variations in the deep water column (below 250 m) indicate active energy transfer there (Figs. 1 and 3). In a quiescent deep monimolimnion of a 600 m deep meromictic lake Matano, for example, temperature profiles in the deep waters remain constant within one hundredth of a degree (instrumental accuracy) over decades [19]. In contrast, in Lake Kivu the deep water temperature profiles vary significantly over time (Fig. 1), and possibly laterally among deep sampling locations. Some lateral variability may be expected in the vicinity of subsurface inflows (e.g., Fig. 4); however, the time series in Fig. 3 also indicates significant temporal variability. This suggests that the deep monimolimnion experiences water movements and heat fluxes, perhaps in response to localized heating or seismic events. For example, our temperature recorder at 350 m registered two anomalous events in a six-month period (Fig. 3) that were not obviously correlated to the records higher up in the water column. The mixing intensity in the deep waters therefore cannot be assumed on long time scales to be close to molecular diffusion, as assumed by previous models (e.g., [8], [22]) or inferred from measured temperature microstructures [15]. Vertical mixing in Lake Kivu remains poorly quantified, as standard methods are difficult to apply under the nearly steady-state conditions [19] and the effects of episodic events are difficult to take into account. Past modeling approaches, in particular, assumed a constant eddy diffusion coefficient between the major pycnoclines [8] or attempted calculating the mixing coefficients from observed double-diffusion structures [12], [16].

**Figure 6:**
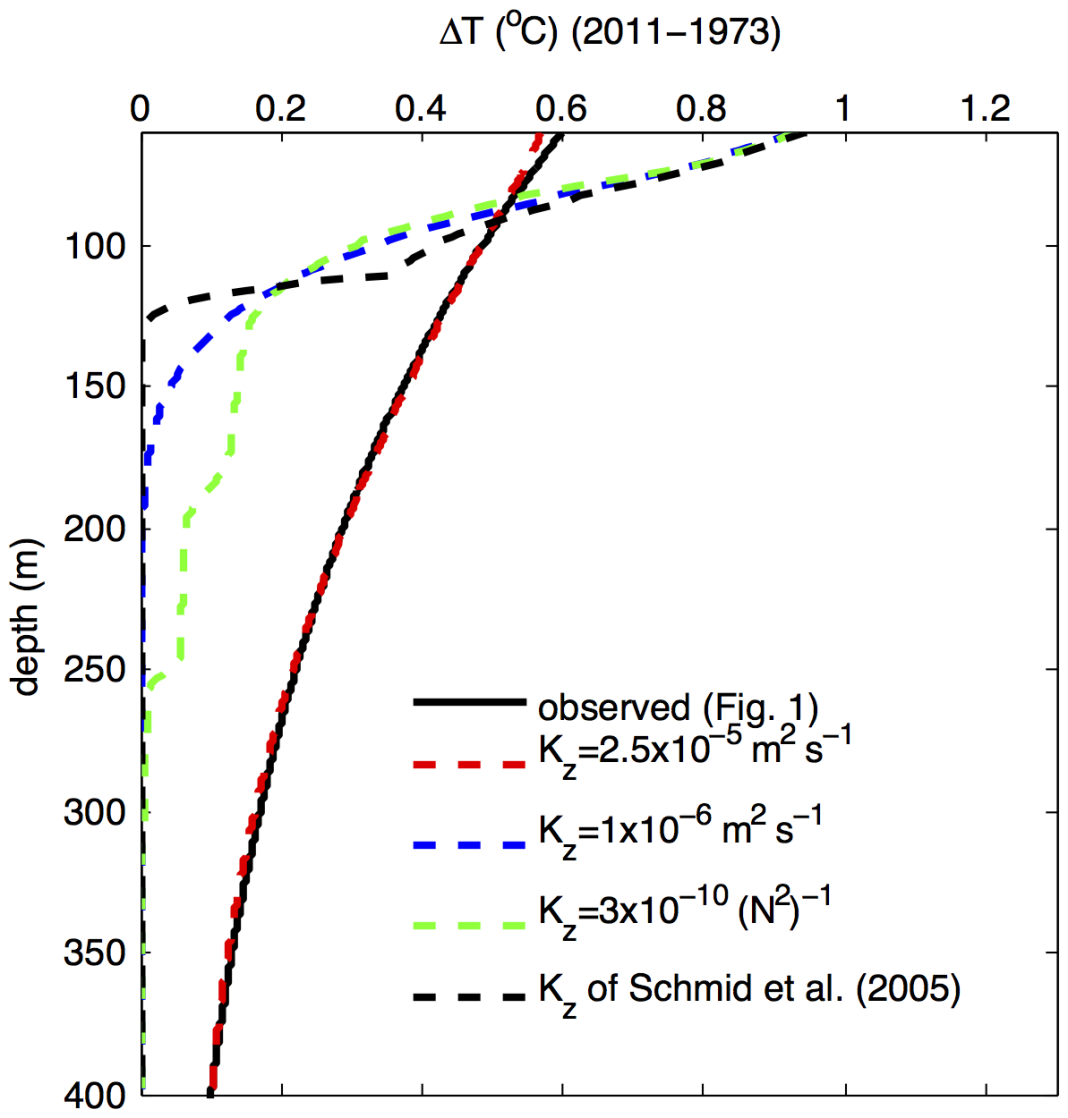
Simulated vs. observed change in water temperature in response to decreased epilimnetic heat removal. The change in water temperatures between 1973 and 2011 is simulated for a decrease in heat removal at the base of the mixolimnion of 0.5 W m^−2^. The solid black line is the same as the exponential fit line in Fig. 5. Dashed lines illustrate the simulation results for different values of the mixing coefficient K_z_: selected constant values, values obtained from the correlation of K_z_ with the stability frequency N_2_ as suggested in [19], and tabulated values suggested for Lake Kivu in [8]. For comparison, the molecular thermal diffusivity in water (at 25°C) is 0.143⋅10^−6^ m^2^ s^−1^.

The warming rate that is greater in the upper than deep monimolimnion (Fig. 4) suggests that the lake has been warming primarily from the surface. The effects of surface vs. deep heat sources can be illustrated with a simple model that uses assumed coefficients of vertical mixing (e.g., as suggested by previous estimates [8]) to propagate excess heat (*ΔT*) from the epilimnion downward. For the water of temperature *T*, the evolution of heat content *cρT*, where *c* is the specific heat capacity and *ρ* is the density of water, can be described by a reaction-diffusion equation:
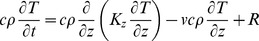
(1)where *K_z_* is the vertical (turbulent) diffusion coefficient, *v* is the vertical advection velocity, and *R* is the rate of heat production at depth *z*. Assuming that for some temperature distribution *T*(*z*) the production of heat in the monimolimnion *R(z)* can be balanced by the heat removal through diffusion and advection (a steady state), the propagation of a disturbance to that state (excess heat) can be described by replacing *T(z)* with *T*(*z*)+*ΔT*(*z*,*t*) and rewriting eq. (1), taking into account that for a steady state *T*(*z*) the right-hand-side of eq. (1) is zero:
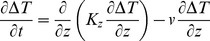
(2)As the diffusion equation (1) is linear with respect to temperature *T* (neglecting the temperature dependencies of *c* and *ρ*), the propagation of the excess heat *cρΔT* (eq. 2) does not depend on the direction of the temperature gradient, which simplifies the simulations. For advective velocities in Lake Kivu (inferred previously to be on the order of 1–8 meters per decade; *above* and in [8]) the advective term in eq. 2 is small in comparison to mixing by turbulent diffusion and can be neglected. Equation 2 was solved using the numerical solver of the water-column module of Aquasim, a software package designed to solve this type of equations in aquatic environments [28]. As a conservative estimate, an increase in heat fluxes of 0.5 W m^−2^ over the steady state was specified at the model's upper boundary, at the base of the mixed layer, and the resultant changes in water temperature *ΔT* were calculated for a forty-year time interval. The results from this simple model (Fig. 6) indicate that the observed warming below 250 m cannot be explained by the propagation of heat from the overlying waters using the currently assumed transport rates (e.g., the eddy diffusion coefficients used in [6] and [8] or the transport rates on the order of thermal diffusivity of water). Either the effective (long term) heat transport rates are higher by an order of magnitude, or the intensity of the subsurface heat sources is increasing (over a hypothetical equilibrium regime where the generated deep heat can be removed by the existing temperature gradients). An alternative way of looking at this is to calculate the characteristic length scale of diffusion 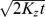. Using the diffusion coefficient of 1×10^−6^ m^2^ s^−1^, the maximum value used in previous modeling studies [8], suggests that after *t* = 40 years the profiles of *ΔT*(*z*) should attenuate over the characteristic length scale of 50 m, whereas observations (Fig. 5) suggest the length scale for exponential attenuation on the order of 190 m. Comparing the diffusive transport of conservative tracers, such as Na, to their removal rates from the epilimnion with the outflow [22], nevertheless, suggests that the vertical transport rates are unlikely to have been significantly underestimated in past studies (M. Schmid, pers. comm.), at least for the upper water column and assuming negligible coastal upwelling. The intensification of the subsurface heat sources thus appears to be a more likely reason for the observed deep warming.

Besides the obvious hydrothermal heat inputs, which may or may not be stable over decadal time scales considered here, warming in Lake Kivu may result from biogeochemical reactions, as originally proposed in [29]. Formation of methane from carbon dioxide and hydrogen, the process responsible for estimated 65% of methane production below 260 m [6], liberates 60 kcal/mol (the standard enthalpy of reaction ΔH_0_ = 240.2 kJ/mol). Acetoclastic methanogenesis releases about the same amount of energy (ΔH_0_ = 245.2 kJ/mol [30]). Based on comparison to 1955 measurements [31], a 1973 study [29] suggested a growth of the methane reservoir by about 1 percent per year, and a 2005 study [8] similarly claimed the increase of about 15% between 1970s and early 2000s. If methane concentrations indeed increased in the past 30 years by 3 mmol L^−1^, formation of this amount of methane would release about 180 cal/L, enough to heat the water by 0.18 degrees, which would be consistent with the temperature change in Fig. 5. Some energy may go towards satisfying the energy requirements of organisms rather than converted to heat, but with the biomass likely being approximately constant over decadal time scales this fraction, which is difficult to estimate, is probably small. Similarly, if the concentrations of CO_2_ also increased by ∼15% (∼13 mmol L^−1^), the heat of dissolution (ΔH_0_ = −19.3 kJ/mol) could further increase the temperature by 0.06 degrees.

## Conclusions

Lake Kivu has experienced significant warming over the course of the 20^th^ century, with temperatures rising over the entire water column. The warming rate in the seasonally mixed epilimnion is similar to those reported in other East African lakes. This rate has likely increased over the past several decades, and is most probably linked to regional climate warming. The warming rates in the deep monimolimnion suggest that heat inputs from deep subterranean sources are not balanced by the removal of heat towards the lake surface. Unlike other deep African lakes where warming increases physical stability through increased temperature gradients, the salinity-stratified Lake Kivu is unlikely to experience strong changes in stability. Nevertheless, continued deepening of the temperature minimum will lead to rarer events of heat removal by epilimnetic mixing, which will further decrease the removal of heat from the deep waters. The warming rate is thus expected to accelerate. The warming trend, the descent of the temperature minimum, and the upward movement of thermoclines all indicate that over decadal time scales the stratification in Lake Kivu cannot be considered to be at steady state.
